# Cross-cultural dataset for the evolution of religion and morality project

**DOI:** 10.1038/sdata.2016.99

**Published:** 2016-11-08

**Authors:** Benjamin Grant Purzycki, Coren Apicella, Quentin D. Atkinson, Emma Cohen, Rita Anne McNamara, Aiyana K. Willard, Dimitris Xygalatas, Ara Norenzayan, Joseph Henrich

**Affiliations:** 1 Department of Human Behavior, Ecology, and Culture, Max Planck Institute for Evolutionary Anthropology, Deutscher Platz 6, Leipzig 04103, Germany; 2 Department of Psychology, University of Pennsylvania, Solomon Laboratories, 3720 Walnut Street, Philadelphia, Pennsylvania 19104-6241, USA; 3 School of Psychology, University of Auckland, Human Sciences Building—East, Level 5, Room 201E-523, 10 Symonds St, Auckland 1010, New Zealand; 4 Max Planck Institute for the Science of Human History, Kahlaische Strasse 10, Jena D-07745, Germany; 5 Institute of Cognitive and Evolutionary Anthropology, University of Oxford, 64 Banbury Road, Oxford OX2 6PN, UK; 6 Department of Psychology, University of British Columbia, 2136 West Mall, Vancouver, British Columbia, Canada V6T 1Z4; 7 Culture, and Development Laboratory, Department of Psychology, The University of Texas at Austin, 1 University Station #A8000, Austin, Texas 78712-0187, USA; 8 Department of Anthropology, University of Connecticut, 354 Mansfield Road Unit 1176, Storrs, Connecticut 06029, USA; 9 Interacting Minds Centre, Aarhus University, Jens Chr. Skous Vej 4, build. 1483, Aarhus DK-8000, Denmark; 10 LEVYNA, Masaryk University, Brno 60200, Czech Republic; 11 Department of Economics, University of British Columbia, 2136 West Mall, Vancouver, British Columbia, Canada V6T 1Z4; 12 Department of Human Evolutionary Biology, Harvard University, 11 Divinity Ave, Cambridge, Massachusetts 02138, USA

**Keywords:** Human behaviour, Cultural evolution

## Abstract

A considerable body of research cross-culturally examines the evolution of religious traditions, beliefs and behaviors. The bulk of this research, however, draws from coded qualitative ethnographies rather than from standardized methods specifically designed to measure religious beliefs and behaviors. Psychological data sets that examine religious thought and behavior in controlled conditions tend to be disproportionately sampled from student populations. Some cross-national databases employ standardized methods at the individual level, but are primarily focused on fully market integrated, state-level societies. The Evolution of Religion and Morality Project sought to generate a data set that systematically probed individual level measures sampling across a wider range of human populations. The set includes data from behavioral economic experiments and detailed surveys of demographics, religious beliefs and practices, material security, and intergroup perceptions. This paper describes the methods and variables, briefly introduces the sites and sampling techniques, notes inconsistencies across sites, and provides some basic reporting for the data set.

## Background and Summary

As the evolutionary sciences of religion and cooperation mature, there is a greater need for rich, comparative ethnographic and cross-cultural psychological research that draws on evidence going beyond samples of university students, or dated, qualitative ethnographic or state-based datasets to test hypotheses^1–10^. Our international team came together to design and execute a large cross-cultural study to examine a range of contemporary concerns in the evolutionary and cognitive sciences of religion, with particular focus on whether and how religion contributes to the expansion of prosocial behavior. Dubbed the ‘Evolution of Religion and Morality Project’, this team also sought to provide the foundations for similar, future research and ensured that data, methodological protocols, and analytical scripts are publicly available to researchers. A report utilizing this data set was subsequently published in *Nature*^11^. Over the next few years, we will continue expanding the dataset to include more variables, more sites, and more experimental conditions. The current available datasets include a sizable portion (591 participants x 86 variables) of the first wave of data collection that occurred during the summer months of 2013.

Emerging evidence suggests that—together with other important factors such as moral emotions, institutions, norms, and markets—some elements of religion can alter the evolutionary trajectory of human cooperation^12–17^. Psychologically, three aspects of religious cognition appear to play an important role in religious prosociality: (1) perceptions of supernatural monitoring^18–20^, (2) supernatural punishment^21–25^, and (3) gods’ concerns^26–28^. Cross-cultural research continues to find an association between ‘moralistic high gods’ and societal complexity and a variety of ecological factors ranging from water scarcity and environmental harshness to animal husbandry^1,3,8,9,29,30^. As societies’ population sizes expand, anonymity makes it easier to defect on norms of prosocial behavior. It is therefore a challenge to maintain such grand-scale social cohesion. In order to develop and stabilize social complexity, then, a host of factors found in human psychology, cultural traditions, and institutions must be in place^31–33^. We predicted that certain religious beliefs—morally concerned, punitive, and omniscient deities in particular—are one such cluster of important factors that may have contributed to and sustained large-scale cooperation. In other words, growing up in a tradition where a god has some knowledge of what you do and think, cares about how you treat other people, and punishes you for mistreating others will inculcate a broader sense of impartiality towards greater numbers of people. The more individuals are willing to engage in stable cooperative relationships, the more likely religious and other beliefs and behaviors are transmitted, shared, and become more widespread. Cultural evolutionary theory predicts that this process is gradual, and that increasingly punitive, moralistic, and knowledgeable gods will coevolve with and contribute to the expansion of fairness and moral behavior toward coreligionist strangers^14,23,34^. We predicted, therefore, that people are more likely to ‘play fairly’ towards coreligionists beyond their immediate community when they claim their moralistic gods know and punish people for treating others immorally. The evidence suggested that this indeed was the case^11^.

In this paper, we define all of the variables included in our previously published study. Note, however, that the dataset also now includes individual items used in scales and additional variables for further exploration. Given the diverse traditions and lifeways of our sample, we envision that this data will be important for testing other demographic, psychological, cultural, and economic hypotheses. We draw much of this article from the Supplementary Materials of our recent report in *Nature*^11^ where readers can find further details and analyses.

## Methods

Our study consisted of a cluster of modules. Study protocols are publicly available online for reuse and examination (http://www.hecc.ubc.ca/cerc/the-cultural-evolution-of-prosocial-religions/the-cultural-evolution-of-prosocial-religions-protocols/). This package includes all original materials distributed to the research teams, including spreadsheets, interview scripts, consent forms, and other materials. All protocols were translated into local working languages of our field sites, back-translated into English, and edited for consistency and clarity.

**Figure 1:**
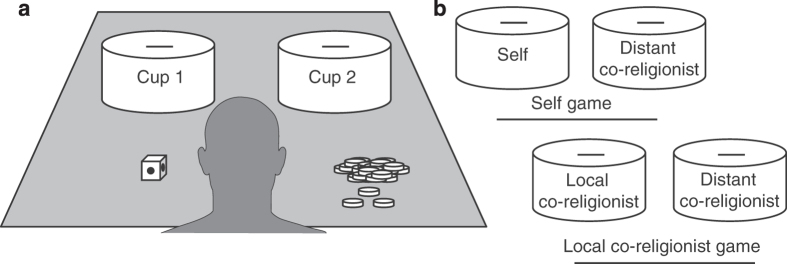
Random allocation game setup. (**a**,**b**), Generic game setup (**a**) and variants used in present work (**b**). Reproduced from Purzycki, *et al.* (2016), *Nature, 530*(7590): 327–330.

This data set includes two of the modules: data from a behavioral economic experiment and survey questions. The behavioral experiment we employed was a modified version of the Random Allocation Game^24,35^. In this game, participants play with two cups, a fair two-colored die, and a stack of coins (in this case, 30 per game) in front of them. Reproduced from our report in *Nature*, Fig. 1 illustrates the basic game set-up. Each cup is designated for some specifically defined individual. Participants must think of which cup they would like to put a coin into, then roll the die. If the die comes up one color, participants are supposed to put the coin into the cup they thought of. If it comes up another color, then they are supposed to put the coin into the opposite cup to the one they thought of. Participants knew that they would be able to keep for themselves the money allocated to their cup, and that we would give the money left in the other cups to the appropriate people. Participants play alone with no other observers present, so if they are inclined to break the rules, they can. Binomial logistic regression analyses can detect which variables have effects on the chances that coins go into any given cup.

In our study, all participants played games first, then answered follow-up questions. Participants played two games: the *Local Co-Religionist Game* and the *Self Game*. The player dyads for each game were as follows. For the *Local Co-Religionist Game*, cups were for: (1) an anonymous co-religionist from participants’ own communities and (2) an anonymous co-religionist from a geographically distant place where participants are not likely to go. For the *Self Game*, cups were reserved for: (1) participants and (2) a different individual from the same community as the distant co-religionists from the other game. Co-religionists were defined as people who share similar beliefs and practices revolving around the deity that most closely approximated to that of the most morally concerned, punitive, and omniscient deity (see below). Participants played these games in randomly assigned, counterbalanced order. Note that in the Tyva Republic, participants played three games (local co-religionist versus participants), but data for those games will be available elsewhere.

Total combined stakes for games were set at roughly a single day’s wage in the local community (i.e., per die roll amounts were the around the average local daily wage was divided by number of games played divided by 30 coins). Show-up fees were approximately 25% of a local average day’s wage. With the exception of the Hadza, all sites used cash. As the Hadza were the least market-integrated sample we had, and cash has inconsistent significance and utility across Hadza groups, the Hadza played with tokens worth 8 oz. (~226.80 g) maize for each roll.

As discussed in more detail below, in five of our sample sites, participants were randomly assigned to a treatment or control condition in order to more confidently assess causation between religious symbolism and cooperation. Treatment conditions were items or a location of religious significance.

### Participants

**Figure 2:**
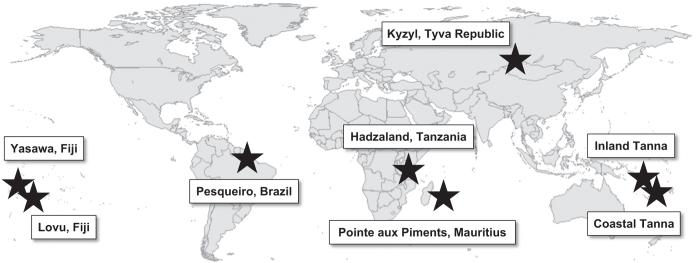
Map of eight field sites included in data.

We executed this study in eight different field sites: (1) Coastal and (2) Inland Tanna, Vanuatu; (3) Tanzania among the Hadza, (4) Lovu (Indo-Fijians) and (5) Yasawa (Native Fijians), Fiji, (6) Pointe aux Piments, Mauritius, (7) Pesqueiro, Marajó Island, Brazil, and (8) Kyzyl, Tyva Republic, Russia (*N*=591; M_age_=37.32; s.d.=14.91; 310 females). We recruited participants using various sampling techniques (e.g., randomly selected from censuses, sampling entire camps, and door-to-door recruitment). The following brief introductions of the field sites include descriptions of site-specific sampling procedures. Figure 2 (adapted from the *Nature* report) illustrates the locations of our target subsamples.

### Coastal and Inland Tanna Island, Vanuatu

Traditionally, residents of Tanna Island in Vanuatu have been swidden horticulturalists although a market-based economy plays an ever-increasing role throughout the island^36^. Religion is a syncretic mix of Christianity and the traditional ‘*Kastom*’ beliefs and practices, as well as millenarian ‘cargo cults’^37,38^. Atkinson led the collection of data at two sites on Tanna: a cluster of three inland, predominantly *Kastom* hamlets that rely almost exclusively on subsistence farming for food production, and a wealthier coastal, Christian village in which home production accounts for about two thirds of food consumption. In the coastal village, the Moralistic God was the Christian God and the ‘Local God’ was *Tupunus*, a local spiritual force associated with garden magicians. In the inland hamlets, the Moralistic God was the *Kastom* creator god and culture hero, *Kalpapen*, and *Tupunus* was also selected as the Local Deity. For The Coastal sample, the distant co-religionist cup was reserved for ‘someone from another Christian village’ whereas the distant co-religionist for the Inland sample was ‘someone from another *Kastom* village’. For the Coastal participants, the study was conducted in the Bislama language, while the study was conducted in Navhal for the Inland sample.

At both sites on Tanna, recruitment followed an initial community meeting where the team explained their wish to run the economic games and interviews to learn about life on Tanna and how people make decisions about money. At the meeting, the team explained that participation was entirely voluntary and those participating would receive a small amount of money. The Kastom site on Tanna comprised three hamlets with a population of 90–100 adults. Everyone over the age of 18 in each of the three hamlets was given the opportunity to participate. In an effort to minimise the possibility of collusion, the economic game was played over 4 days—one day per hamlet, with a third day for extras who were not available on the game day. In total, 82 individuals were recruited to participate. Six of these were excluded because they failed the comprehension questions. The Christian site on Tanna was a coastal village of approximately 200 adults. 44 participants were recruited via sampling from a recent census of households in the village. Games were run over 2 days. It was not possible to sample completely randomly because some participants did not show up on game day. However, the team attempted to recruit participants from households across the entire village.

### Hadzaland, Tanzania

The Hadza are hunter-gatherers who largely subsist on wild game, fruits, tubers and honey in the savannah woodlands of western Tanzania. Some have reported the Hadza to be only minimally religious^39^ but this appears to be changing. The majority of Hadza (~80%) of a previously assessed sample claimed to believe in the existence of *Haine*—a celestial based supernatural agent. However, of that sample many neither claimed to know nor think *Haine* had supernatural capabilities. Approximately half of this sample believed in both *Ishoko* (another celestial being) and *Haine*, but most of those individuals thought these two beings are the same god. Although many Hadza incorporate *Ishoko* into their belief of *Haine*, *Ishoko* on its own usually refers to just the physical sun while *Haine* may refer to the moon. The distant co-religionist cup in the behavioral experiments was reserved for another Hadza person living at an unspecified camp. The study was conducted in both Hadzane and Swahili for the Hadza.

Apicella and three research assistants visited nine different Hadza camps around the eastern side of Lake Eyasi, Tanzania. Some camps, particularly those in the southeastern side of the lake, are more remote. It was in this area where Apicella and her team began their work. To find the first camp, her team visited locations where the Hadza have been known to set up camp in past years. After happening upon the first camp, individuals in the camp directed her to the next nearest camp. Sampling of camps continued in this fashion—not unlike a snowball or chain sampling technique. Occasionally, a Hadza participant would accompany the researchers in their vehicle to help locate the next camp. In each camp, all Hadza estimated to be over 18 years of age and present in camp were eligible to participate in the study.

### Lovu, Fiji

Indo-Fijians are a diaspora population brought to Fiji from India by the British as indentured workers^40–42^. Wage labor is the primary source of income but Indo-Fijians also farm sugar cane. Religiously, Indo-Fijians are primarily Hindus and Muslims though some are Sikhs or Christian. The present sample includes Hindus from Lovu village on the island of Viti Levu. Participants largely claimed that all Hindu gods are different aspects of one single deity, *Bhagwan* and this deity was therefore selected as the moralistic deity for this study. As one could not be identified, no Local Deity was selected. In the experiments, the distant co-religionist was a Hindu living on Vanua Levu, the second largest island in Fiji. For the Indo-Fijian sample, this study was conducted in Fiji-Hindi and English.

Participants came primarily from the villages of Lovu Seaside and Lovu HART. Some additional participants came from the nearby villages of Koro Pita, and Drasa. They were contacted in person at their homes ahead of time and asked if they would like to participate. The Lovu research group obtained names and contact information from those who agreed. Though specific time slots were given to all participants ahead of time, almost no one showed up in their allotted time slot. Because of this, participants were taken whenever they showed up. Since every identifiable Hindu household in Lovu Seaside and Lovu HART were contacted, all participants from those villages were accepted. Only participants from Koro Pita and Drasa who had been previously contacted, or who showed up at the same time as those that had been previously contacted, were accepted. Participants from outside of Lovu who had been told about the experiment by their friends or family members after their friends and family members had participated were not allowed to participate.

### Pointe aux Piments, Mauritius

The island nation of Mauritius lies around 1,200 miles off the coast of southeastern Africa. While it was the last sovereign country on earth to be settled by humans (in the 18th century), today it is one of the most ethnically diverse places worldwide. Previously reliant on a monoculture of sugar cane, Mauritian economy diversified and prospered after the country gained independence from the British in 1968. Today, urban Mauritius has a notably diverse, market-based economy while rural populations rely primarily on horticulture and fishing. Data in this set was collected in Pointe aux Piments, a small village that lacks industry. Residents there primarily fish, cultivate, and serve the tourism industry^43–45^. While Pointe aux Piment is roughly split between Hindus and Christians, the present sample is exclusively Hindu with Shiva functioning as the most popular Moralistic Deity. The Local Deity was a *nam*, a concept similar to a spirit or soul. Distant-coreligionists were Hindus from La Gaulette, a small distant village largely unknown to participants. This study was conducted in Mauritian Creole for this sample.

We recruited Hindus between ages 17–78 years old. The Mauritius research group used a convenience sampling technique whereby local assistants positioned in a host of locations around the village randomly invited people passing by to take part in the study. This allowed us to enroll many participants in a matter of a few days, thus minimizing collusion. Informal post-hoc analyses suggested that this sample was similar to the general village population based on comparisons of basis demographic variables of our sample to those of the latest census^44^.

### Pesqueiro, Marajó Island, Brazil

At the mouth of the Amazon River lies Marajó Island, Brazil. Pesqueiro is a small fishing village on the east side of Marajó Island. Residents of Pesqueiro rely primarily on fish sales and tourism. Most residents are Catholic, although some are Evangelical Protestants^46,47^. For this sample, the Moralistic Deity was the Christian God (*Deus*), and Our Lady of Nazareth (*Nossa Senhora de Nazaré*), the region’s patron saint served as the Local Deity. The distant co-religionist was a Christian from Rondon, a distant but familiar town in mainland Pará state. For residents of Pesqueiro, the study was conducted in Portuguese.

Participants were sampled from the entire village. An up-to-date census of the entire population (total: 309; 92 families) was obtained and all adults were included for random selection. Individuals were approached in their homes and invited to take part in the study. If unavailable, an alternative was selected from a reserve list (also randomly generated). Thirty-four out of a total 128 individuals were unavailable on the scheduled date for the study, leaving a total of 94 scheduled participants, who were randomly assigned to conditions. Fourteen people did not show for their session.

### Kyzyl, Tyva Republic

Hailed informally as the geographic centre of Asia, the Tyva Republic lies in southern Siberia, just north of the western portion of Mongolia. Urban Tyvans subsist primarily on a market-based economy while rural Tyvans herd sheep, goats, cattle, and/or yaks^48^. This sample was drawn exclusively from the capital city of Kyzyl. Most Tyvans identify as Buddhist, but also engage in religious practices associated with shamanism, animism, and totemism. Buddha-Burgan (‘Buddha God’) functioned as the Moralistic Deity, while an unspecified *cher eezi*, or ‘master of the place’, a spiritual lord over resources and regions^25,49,50^ functioned as the Local Deity. The distant co-religionist was from Ak Dovurak, a familiar asbestos-mining town about a 4-hours west of Kyzyl by car. All experiments and interviews were conducted in Tyvan, though some did ask for game instructions in Russian for clarity.

The Tyva research group’s efforts to have recruits participate in follow-up sessions were futile. They therefore conducted single sessions, each lasting around 90 min per participant. Four assistants used random, chain and snowball sampling to recruit people who would contact the lead assistant to coordinate meeting places and times. Assistants only divulged that they required up to 90 min of participants’ time and that they would be paid for it. They also encouraged enlisted participants to also recruit more people before their participation, but not after, and they refused all unsolicited candidates. Assistants also asked each participant about all of the information that they knew about the study and everyone conveyed only the allowed information. Assistants recruited people on the basis of their Buddhist and/or Shamanist identification, Tyvan ethnicity, and fluency of the Tyvan language.

### Yasawa, Fiji

Yasawa Island is on the northwestern corner of the Fijian archipelago. Yasawans are primarily fisher-horticulturalists^51–54^. The majority of Yasawans identify as Wesleyan Methodists though a large minority associate with the evangelical Assemblies of God. However, traditional beliefs and practices devoted to ancestor spirits (*Kalou-vu* or ‘root/ancestor god’) continue to thrive. For this sample, the Moralistic Deity was the Christian ‘Bible God’, while the *Kalou-vu* represented the Local Deities. Cups for the distant co-religionists in Yasawa were Fijian Christians from another island. The Yasawan protocols were all conducted in Bauan.

Indigenous Fijian participants were recruited by invitation based upon their location within the village. The games were played in houses across the village, one on each day. Villagers living closest to those houses were invited to attend in waves of eight participants by Indigenous Fijian research assistants who administered the games and post-game interviews.

### Experimental conditions

In five sites—(1) Lovu, Fiji; (2) Pointe aux Piments, Mauritius; (3) Pesqueiro, Brazil; (4) Kyzyl, Tyva Republic; and (5) Yasawa, Fiji—participants were randomly assigned to a treatment or control condition. Treatment conditions consisted of playing the economic game near or in a religious symbol or setting associated with the relatively more morally concerned, punitive, and knowledgeable deities we determined using preliminary ethnographic interviews. Images of the prime conditions are included with the data sets. In the design phase of our project, we agreed to ensure that all conditions lacked explicit indices of agency (e.g., eyes or human forms) in order to control for agency effects found in other studies^55–58^. We expected that treatments would harness the prosocial effects of religious beliefs across sites that used them; religious symbols ought to have caused less bias in coin allocations. Across a wide variety of model specification, initial analyses^11^ using condition as a fixed effect showed no overall, across-site effect on coin allocation in the game. This may be due to treatments’ differential interactions and effects across sites.

In Lovu, Fiji participants in the treatment condition played near a small lingam (~20 cm tall) with a small trident wreathed in an orange garland. A lingam is an upright cylindrical stone sitting on a rimmed disk (*yoni*). The lingam and trident sat on a short covered table (~30 cm tall) placed in one of the two experimental areas. These items were chosen because they are well-recognized abstract symbols of the god Shiva. Unlike most representations of deities in Fiji-Hinduism, they are not human-like and do not have eyes. Participants in the control condition played without any religious imagery or symbolism present.

The Mauritius research group used contextual primes in the form of two rooms, one of which was part of a Hindu temple and one of a secular one (a restaurant). The two locations were in the same neighborhood and had similar size, but different functions and associations. The secular location was rented for the duration of the experiment, while the religious location was used by permission from the temple authorities.

Brazilian participants in the treatment condition played games near an open Holy Bible and a necklace with a wooden cross pendant placed on the game table approximately half a meter from the seated participant. The necklace and pendant were placed over the text. The items were selected on account of their familiarity and broadly equivalent religious significance to Catholic and Evangelical participants alike. The control group played in the absence of any religious paraphernalia.

Tyvans in the treatment condition played games with a Buddhist luck charm (*kamgalal*) placed in front of the cups. Tyvans typically use such charms for protection, typically in homes or in vehicles. In post-game interviews, participants often claimed these charms attract wealth and good luck, as well as warding off evil spirits. The charm’s design represents the Dharma wheel (*dharmachakra*), a central symbol in Buddhism. The control group played without the charm.

For our native Fijian subsample from Yasawa, participants played games on top of dark or navy blue *sulus* or cloths with various patterns printed on them. In the control condition, the *sulu* had a flower and the text ‘*Bula Fiji*’ or ‘Life/Hello Fiji’ printed on it and in the treatment condition, the *sulu* had a cross and Bible printed on it with the following Bible verse: ‘Jesus said, ‘All things are possible to him who believes’ Mark 9:23’.

## Data Records

### Data sets

There are often complications associated with working with samples unaccustomed to entertaining certain forms of questions such as open-ended questions or Likert-scales. In our case, we accommodated the hunter-gatherer Hadza and as such, we altered many of the questions and answer options for them. We therefore assembled two data sets posted on Harvard Dataverse. One (Data Citation 1) includes all of the original Hadza data for the sake of posterity (see ‘Data Notes’ section below for specific items). Using this data set will create problems as the data points are saved as text rather than numerical values. We therefore also include another set (Data Citation 2) which is identical, but removes the values of those altered or suspect questions. Included in these entries are: (1) xls files that include codebooks with variable definitions; (2) files in csv format; (3) R scripts for analyses; and (4) images of the experimental treatment conditions.

### Code availability

Code and scripts for analyses in R are available with our data sets as well as on the project website: http://www.hecc.ubc.ca/cerc/the-cultural-evolution-of-prosocial-religions/the-cultural-evolution-of-prosocial-religions-protocols/.

For the sake of illustration and convenience, we provide the following truncated version of the R code for the experimental data.

RAG1.E <-glm(cbind(COREL.1, INGROUP)~

DIEPUN + LGDIEPUN + OMNI.BG + OMNI.LG +...

as.factor(HONEST)+ as.factor(TREATMENT)+ as.factor(INGFIRST)+

relevel(SITE, ‘Tyva Republic’)

data = cerc, family = binomial)

Here, the dependent variable is the concatenated (cbind) cups from the *Local Co-Religionist Game*. Analyzing the *Self Game* simply requires replacing COREL.1, INGROUP with COREL.2, SELF. This code includes some religiosity variables for the sake of illustration, but also the game variables HONEST, TREATMENT, and INGFIRST. As participants from the Tyva Republic had allocations closest to what we would expect if people played fairly, we used them as our reference group. This is made possible by the relevel(SITE, ‘Tyva Republic’), component of the code. The dataset in this case is simply the object ‘cerc’. And finally, as the distribution of the allocations should have followed a binomial distribution (30 Bernoulli trials), we used the binomial link option.

The general code structure in STATA is as follows:

blogit CUP1 SUM1 variable1 variable2 … variablen, robust cluster(SITE) or

Here, ‘blogit’ instructs the software to run a binomial logistic regression. Unlike R which concatenates the two cups, STATA requires one target cup and a variable including all possible values (i.e., SUM1 always equals 30 in our data). The command robust cluster(SITE) instructs STATA to generate, robust clustered standard errors by field site and the or command calculates odds ratios.

### Variable definitions

This data set consists of three general types of variable. One type consists of sample variables—metadata of the groups and researchers involved. Another type is the experimental game data. Finally, it also includes survey data that we solicited from each participant in the game. This survey data consists of: demographic variables, measures of supernatural beings’ characteristics, and measures and evaluations of subjective intergroup relations. Note that all missing values are defaulted to ‘NA’ for immediate use in R. In the following, all variable names in bold correspond to the variable name in the data set.

### Sample variables

Sample variables include identifiers for participants, their location, and the researchers leading the project in those locations. **CERCID** is a unique identification code for each participant (N=591). **RESEARCHER** identifies which researcher on the team led the data collection in each **SITE** (see Table 1). This dataset includes data from eight different field sites collected by seven different field researchers and their teams.

### Experimental data variables

Experimental data variables include condition, order and outcome of games, and participants’ thoughts on the games. **TREATMENT** denotes whether (=1) or not (=0) participants played with a religious prime of various sorts. **ORDER** is a factor variable that notes the order in which participants played games. In this code, the *Local Co-Religionist Game* is denoted with a ‘1’ and the *Self Game* is denoted with a ‘2’. So, if participants played the *Self Game* first, **ORDER** would read ‘21’. Note that in the Tyva Republic, participants played three games (a LOCAL versus SELF game). **INGFIRST** dummy codes whether (=1) or not (=0) participants played the *Local Co-Religionist Game* first.

**COREL.L** and **INGROUP** represent the number of coins participants placed in each cup for the *Local Co-Religionist Game* while **COREL.S** and **SELF** are the cups in the *Self Game*. **SUM1** and **SUM2** all, therefore, add to 30 as these are the sums of coin amounts in both cups per game. This variable is particularly useful for analyses in STATA and SPSS. Note that in the *Local Co-Religionist Game*, there are only 589 participants rather than 591 in the *Self Game*. We removed one individual from the Coastal and Inland Tanna sites, because coins were visible in the cups for those games.

Among the questions we asked upon completion of the game, we asked participants what they thought the game was about. If they mentioned ‘honesty’, ‘fairness’, or ‘cheating’ in their responses, they were given a score of ‘1’ for the **HONEST** variable, and a ‘0’ for all other responses.

### Demographic variables

Demographics include standard individual-level demographic data, but also include measures of subjective material security. Participant **SEX** is coded in the standard fashion (0=female; 1=male). **AGE** is the reported age of participants while **AGE.C** is the centered-at-sample-mean age value. **FAMILY** records family status using the following codes: 1=single; 2=married; 3=engaged; 4=divorced; 5=widowed. Six individuals from Pesqueiro, Brazil noted ‘amaziado’ (lit. living together) as their status. We recommend that these be recoded as ‘1’ for single, but retain them for posterity.

We also asked how many **CHILDREN** participants have fathered or given birth to. **FORMALED** represents total years of formal education. As many participants have had no formal education, we do not recommend centering these variables by the sample mean. **HOUSEHOLD** represents the number of people individuals reported in household. Note, however, that we are unsure about whether or not people counted themselves as we did not qualify the question. Note, too, that when asked about household size, one individual in the Tyvan sample said ‘Always three people, but around 15 coming and going’. This was converted to three.

As fluency of native language is often a marker of education, class, and political affiliation and can affect gameplay, we asked participants to rate their fluency levels of their ethnically native language (**NATLANG**). These were on scales of 0 to 4: 0=I don’t speak [the language]; 1=not well; 2=well; 3=very good; 4=fluent.

In order to measure participants’ subjective sense of material security, we asked them eight questions about insecurity and security at various time scales. To measure material insecurity, we used the following frame: *Do you worry that in the next _________ your household will have a time when it is not able to buy or produce enough food to eat?* For material confidence, we used: *How certain are you that you will be able to buy or produce enough food to eat in the next _________?* We asked four questions per frame that varied by time period: a) month, b) six months, c) year, and d) five years. These are denoted as **MAT1**-**4** (insecurity) and **MAT1C**-**4C** (confidence) in the data set. **MMAT** is the mean value of the material insecurity questions and **MMATc** is the mean of the material confidence questions.

### Measures of gods’ characteristics

Prior to playing games, we conducted a preliminary set of interviews about the local religious landscape. From these interviews, we selected two locally salient deities: ones that most approximated to the most morally concerned, omniscient, and punitive deities and ones that were locally important or salient, but not as obviously concerned with morality, knowledgeable, or punishing. We designed questions for experimental participants based on these deities. Note that no obvious candidate local deity was identified among the Indo-Fijians (Lovu), and as such these questions were not asked. All of these variables have the same root name, but vary by the initials ‘**BG**’ (for ‘Big Gods’^14^) and ‘**LG**’ (or ‘Local Gods’).

We asked questions about both deities in randomly assigned, counterbalanced order (**BGLG1ST**: 0=local deity questions asked first; 1=moralistic god questions asked first). To measure beliefs in gods’ punishment and knowledge breadth, we created two questions per domain. For punishment, we asked two dichotomous (no=0, yes=1) questions:

- *Does ______________ ever punish people for their behavior?* (**BGPUNISH/LGPUNISH**)
- *Can _________ influence what happens to people after they die?* (**BGDIE/LGDIE**)

**DIEPUN** is mean value of responses to these two questions for our ‘Moralistic Gods’, while **LGDIEPUN** is the equivalent for the relatively less moralistic ‘Local Gods’. **OMNI.BG** and **OMNI.LG** are mean values of the following dichotomous (no=0, yes=1) knowledge questions for the Moralistic God and the Local God respectively:

- *Can _________ see into people’s hearts or know their thoughts and feelings?* (**BGFEEL/LGFEEL**)
- *Can _________ see what people are doing if they are far away in* [a distant town or city familiar to locals]*?* (**BGSEE/LGSEE**)

We also aggregated the mean of these four questions into our **HIGHGOD** variables for the Moralistic (**HIGHGOD.BG**) and Local (**HIGHGOD.LG**) Gods. Many participants among the Inland Tannese and Hadza responded that they simply did not know the answer to these questions even though this was not a formal option for responses. We therefore noted where this was the case in the dummy variable **DKDIEPUN** (1=’I don’t know’).

To measure the degree to which participants thought their deities were ‘moralistic’, we asked three questions per deity about *How important is it for ___________ to punish…* for: theft (**BGSTLIMP/LGSTLIMP**), lying (**BGLIEIMP/LGLIEIMP**), and murder (**BGMURDIMP/LGMURDIMP**). Responses were on scales of 0 to 4: (0) Not important at all; (1) A little important; (2) Important; (3) Very important; (4) The most important. **MBG** is the mean score for these items for the Moralistic God whereas **MLG** is the equivalent for the Local God. We also asked questions with the same moral infractions, but measuring the frequency in which deities punish for such behavior (**BG/LG STEAL**, **LYING**, or **MURDER**). Response options were: (0) Very rarely/never; (1) A few times per year; (2) A few times per month; (3) A few times per week; and (4) Every day or multiple times per day. As the Hadza had difficulty with scales, these were converted to dichotomous questions (e.g., ‘Does Haine punish people for stealing?). As such, in one data set, we include responses for the Hadza stored as text for posterity, but also include a duplicate set without this data for ease of analysis.

We also asked: *How often does_________ assist people in their lives or reward them for proper behavior?* for both gods (**BGREWARD/LGREWARD**). Because of a couple of complications, there are a few important things to note about this variable. Initially, this scale was on a 5-point Likert scale from 0 to 4: (0) Very rarely/never; (1) A few times per year; (2) A few times per month; (3) A few times per week; and (4) Every day or multiple times per day. Participants in Pesqueiro and the Tyva Republic used these scales. However, these options proved to be too awkward for participants (e.g., to say that God rewards people ‘a few times per month’ was strange). We therefore converted these scales to a more standardized 4-point scale: (0) Never; (1) sometimes; (2) frequently; and (3) all the time. The Coastal and Inland Tanna, Lovu, Mauritius, and Yasawa sites used this version. However, for the Coastal Tannese (Bislama language), this scale suffered from a translation error where ‘frequently’ and ‘all the time’ were virtually indistinguishable. Because of these problems, we divided each individual response by the maximum possible response by site. **BGR1** and **LGR1** include these values for the ‘Moralistic’ and ‘Local’ gods, respectively. **BGR2** and **LGR2** are the same values, but without the Coastal Tanna sample. The Hadza answered the question: ‘*Does _________ assist people in their lives or reward them for proper behavior?*’ with ‘yes’, ‘no’, and ‘I don’t know’ as options.

### Additional religiosity data

The set also includes data from a range of additional religious questions that have not been included in previous reports. Participants (other than the Hadza) answered frequency questions on the aforementioned 0-to-4 frequency scale and dichotomous questions (marked with an asterisk) were answered with either a yes (1) or no (0). Note that the Hadza did not answer (1) using the frequency scale, but answered simpler questions (e.g., ‘Do you think about _________?’) with the options of ‘yes’, ‘no’, and ‘I don’t know’. The Hadza did not answer (3).

1. *How often do you think about _________?* (**BGTHINK**/**LGTHINK**)
*Do you perform activities or practices to talk to, or appease _________? (**BGPERF/LGPERF**)
3. *If yes, how often?* (**BGPERFHO/LGPERFHO**)
4. *How frequently do you worry about what __________________ thinks about you?* (**BGFREQW/LGFREQW**)
*Does__________________ care about how people treat strangers? (**BGSTRANGER/LGSTRANGER**)
*Does__________________ care about how people treat other people who perform rituals for _________? (**BGOTHERRIT/LGOTHERRIT**)
*Does __________________ care about whether people perform certain rituals? (**BGPERFC/LGPERFC**)

### Group relations and evaluations

In order to hold variation in intergroup relations constant, we asked five questions designed to assess subjective thoughts of emotional proximity to various groups. We used a visual Fusion scale^59^ to measure the following:

1. *Using these pictures, how emotionally close do you feel to a DISTANT?* (**CORELEMO)**
2. *Using these pictures, how emotionally close do you feel toward an LOCAL?* (**INGREMO**)
3. *Using these pictures, how emotionally close do you feel toward an OUTGROUP?* (**OUTGREMO**)
4. While they were not the focus of our study, we defined OUTGROUPs as ‘non-co-religionists living in a distant, but known place’. Outgroups (in parentheses) for each site were: Yasawans (Indo-Fijians); Tannese (people from Noumea, another Pacific island outside the Vanuatu archipelago); Lovu (Muslims from Vanua Levu); Hadza (the Datoga, Nilotic pastoralists living nearby); Pesqueiro (Evangelicals or Catholics from São Paulo depending on participants’ affiliation); Tyva Republic (Christian Russians from Ak Dovurak); Mauritius (Muslims in Mauritius).
5. In order to measure participants’ individual sense of the religious similarity between DISTANT and LOCAL co-religionists, we asked:
6. *How similar are DISTANT’s traditions/religious beliefs and practices with the LOCAL?* (**CORELSIM**)
7. also using a visual scale ranging from −2 (very different); −1 (different); 0 (same); 1 (similar); 2 (very similar). Participants could therefore point to the most accurate way they felt. The Hadza did not answer **CORELSIM**. Note, too, that we did not include these data for the Hadza in our original article as their consistent difficulty with scales renders the data suspect. We nevertheless include it in the main data set, but remove it in the minimized set.
8. Previous studies show that in contexts where the participants view the police as effective and responsible, they are more likely to play according to the rules in similar games^24,35^. As such, we asked about how participants felt about the police:
9. *Most members of the police are:* (−2) very bad, (−1) bad, (0) neither good nor bad, (1) **good**, (2) very good (**POLEVAL**)

Note that the Hadza used a simpler evaluation scale for this question: ‘bad’, ‘good’, and ‘I don’t know’. Again, we include data sets that include these data points, as well as one that removes them for ease of omnibus analyses.

### Ethical review board approval

Protocols were initially approved by the University of British Columbia’s Behavioural Research Ethics Board (BREB) and subsequently approved by the equivalent at each individual researcher’s home university prior to execution: Atkinson (University of Auckland, New Zealand); Apicella (University of Pennsylvania, United States); Cohen (University of Oxford, United Kingdom); and Xygalatas (Masaryk University, Czech Republic). McNamara, Purzycki, and Willard were approved through the original application as they were all affiliated with the University of British Columbia at the time of study.

## Technical Validation

### Collusion control and sampling

As each field site posed its own organizational challenges, we employed different sampling procedures per site. Table 1 details the sampling methods used in each site. However, because the game lends itself to external coordination and collusion, we ensured that no unsolicited people participated and participants could not assist with further recruitment. All participants played anonymously and could not interact with others waiting to participate.

In order to ensure that participants understood the game, we asked a series of five test questions before beginning the experimental tasks. If they did not pass, they were allowed their show-up fee, but not considered for the study. Only six individuals from Inland Tanna failed to pass test questions and were subsequently deleted from the sample. All participants in the present data set passed the test questions.

### Data audit procedures

Purzycki created spreadsheets with controls on each column for data validation. In the field, researchers and assistants initially recorded all data on paper files and subsequently entered data into the spreadsheets. Upon project completion, researchers submitted spreadsheets as well as the hardcopies or scans of hardcopies of the datasheets to Purzycki who then organized a team of auditors to audit data to check for consistency between hardcopies and spreadsheets. The team consisted of four assistants and McNamara, Purzycki, and Willard who did not audit their own data. The audit team tabulated any inconsistencies between the hardcopies and electronic datasets and reported them to Purzycki who then reported all inconsistencies to the researchers for evaluation, correction, and integration.

### Data notes

We have included two primary data sets. One (CERC_DataSet_HADZA_FULL) includes all of the original Hadza data for all of the questions that were altered due to difficulty with scales. The other (CERC_DataSet_Main) is identical, but removes the Hadza data for all modified questions (**BGTHINK, BGFREQW, BGSTEAL, BGLYING, BGMURDER, BGREWARD, LGTHINK, LGFREQW, LGSTEAL, LGLYING, LGMURDER,** and **LGREWARD**) as well as those that may be suspect given their difficulty with scales (**POLEVAL, CORELEMO, INGREMO,** and **OUTGREMO**).

The online Supplementary Materials for our *Nature* paper include: basic mean and standard deviation reports, a correlation matrix of target variables, construct validity analyses for scales, various tests of difference between the moralistic and local god scales, and a plethora of various models predicting game allocations.

## Usage Notes

This data set contains a variety of domains that would be of interest to explore relationships between demography, religious cognition, inter-group relations, and cooperation. Cultural anthropologists, economists, and psychologists interested in comparative or single-society research can use the data to generate reports about people and traditions that are rarely considered part of mainstream research samples^2,5^. And, given the recent interest in the cognitive and evolutionary sciences of religion^60,61^, this unique and rich data set, as one microcosm of the vast cultural diversity in societal patterns, beliefs, and behaviors, can provide researchers with opportunities to examine contemporary questions and test hypotheses.

## Additional Information

**How to cite this article:** Purzycki, B. G. *et al.* Cross-cultural dataset for the evolution of religion and morality project. *Sci. Data* 3:160099 doi: 10.1038/sdata.2016.99 (2016).

**Publisher’s note:** Springer Nature remains neutral with regard to jurisdictional claims in published maps and institutional affiliations.

## Supplementary Material



## Figures and Tables

**Table 1:** Site, Deities and Economy.

**Site**	**Country**	**Researcher**	**Sampling Method**	**N**	**Main Economy**	**Moralistic Deity**	**Local Deity**	**Prime**
Coastal Tanna	Vanuatu	Atkinson	Cluster sampling (census)	44	Horticulture/ Hunting	Christian God	Garden Spirit (*Tupunus*)	—
Hadza	Tanzania	Apicella	Entire camps	68	Hunting	Celestial Figure (*Haine*)	Sun (*Ishoko*)	—
Inland Tanna	Vanuatu	Atkinson	Entire community	76	Horticulture/ Hunting	*Kalpapan* (Traditional)	Garden Spirit (*Tupunus*)	—
Lovu	Fiji	Willard	Door-to-door	76	Wage Labor	Hindu *Bhagwan*	—	Statue
Mauritius	Mauritius	Xygalatas	Random sampling (street)	94	Wage Labor/ Farming	Hindu Shiva	Spirit/Soul/Ghost (*Nam*)	Temple
Pesqueiro	Brazil	Cohen	Random sampling (census)	77	Wage Labor	Christian God	Virgin Mary	Bible
Tyva Republic	Russia	Purzycki	Random/chain sampling (street)	81	Wage Labor/ Herding	Buddha-Burgan	Spirit-Masters (*Cher eezi*)	Luck Charm
Yasawa	Fiji	McNamara	Door-to-door (cluster)	75	Fishing/ Farming	Christian God	Ancestor Spirits (*Kalou-vu*)	Printed Cloth
